# A recombinant adenovirus-based vector elicits a specific humoral immune response against the V3 loop of HIV-1 gp120 in mice through the “Antigen Capsid-Incorporation” strategy

**DOI:** 10.1186/1743-422X-11-112

**Published:** 2014-06-16

**Authors:** Linlin Gu, Valentina Krendelchtchikova, Alexandre Krendelchtchikov, Robert A Oster, Kohtaro Fujihashi, Qiana L Matthews

**Affiliations:** 1 Department of Medicine, Division of Infectious Diseases, University of Alabama at Birmingham, 845 19th Street South, Birmingham, AL 35294, USA; 2 Department of Medicine, Division of Preventive Medicine, University of Alabama at Birmingham, 1717 11th Avenue South, Birmingham, AL 35294, USA; 3 Department of Pediatric Dentistry, The Immunobiology Vaccine Center, The Institute of Oral Health Research, The University of Alabama at Birmingham, Birmingham, AL 35294, USA; 4 Center for AIDS Research, University of Alabama at Birmingham, Birmingham, AL 35294, USA

**Keywords:** Adenovirus (Ad), Human adenovirus (hAd), “Antigen Capsid-Incorporation” strategy, HIV V3 loop, Humoral immune response, IgG isotype, Neutralization

## Abstract

**Background:**

Due to potential advantages, human adenoviral vectors have been evaluated pre-clinically as recombinant vaccine vectors against several cancers and infectious diseases, including human immunodeficiency virus (HIV) infection. The V3 loop of HIV-1 glycoprotein 120 (gp120) contains important neutralizing epitopes and plays key roles in HIV entry and infectivity.

**Methods:**

In order to investigate the humoral immune response development against portions of the V3 loop, we sought to generate four versions of adenovirus (Ad)-based V3 vectors by incorporating four different antigen inserts into the hypervariable region 1 (HVR1) of human adenovirus type 5 (hAd5) hexon. The strategy whereby antigens are incorporated within the adenovirus capsid is known as the “Antigen Capsid-Incorporation” strategy.

**Results:**

Of the four recombinant vectors, Ad-HVR1-lgs-His_6_-V3 and Ad-HVR1-long-V3 had the capability to present heterologous antigens on capsid surface, while maintaining low viral particle to infectious particle (VP/IP) ratios. The VP/IP ratios indicated both high viability and stability of these two vectors, as well as the possibility that V3 epitopes on these two vectors could be presented to immune system. Furthermore, both Ad-HVR1-lgs-His_6_-V3 and Ad-HVR1-long-V3 could, to some extent escape the neutralization by anti-adenovirus polyclonal antibody (PAb), but rather not the immunity by anti-gp120 (902) monoclonal antibody (MAb). The neutralization assay together with the whole virus enzyme-linked immunosorbent assay (ELISA) suggested that these two vectors could present V3 epitopes similar to the natural V3 presence in native HIV virions. However, subsequent mice immunizations clearly showed that only Ad-HVR1-lgs-His_6_-V3 elicited strong humoral immune response against V3. Isotype ELISAs identified IgG2a and IgG2b as the dominant IgG isotypes, while IgG1 comprised the minority.

**Conclusions:**

Our findings demonstrated that human adenovirus (hAd) vectors which present HIV antigen via the “Antigen Capsid-Incorporation” strategy could successfully elicit antigen-specific humoral immune responses, which could potentially open an avenue for the development of Ad-based HIV V3 vaccines.

## Background

Viral vectors have been engineered for the development of vaccines and gene therapy vectors against types of infectious diseases and cancers [1-3]. Adenovirus (Ad) vectors had accounted for 23.3% of gene-therapy clinical trials by year 2012 [4]. The broad utility of Ad vectors is due to two major advantages. Firstly, the Ad genome is easily manipulated, making replication-defective Ad capable of propagation in complementing cells. Secondly, Ad can infect a broad range of cells, transferring encoded genes and leading to abundant transgene expressions [1,5]. hAd5 has been employed for transgene therapies, whereby encoded genes were inserted in the early regions (E), and expressed under promoters [6-8]. However, 50-90% of normal adults have hAd5 pre-existing immunity (PEI), which immune-clears hAd5 and hampers high levels of transgene-specific immune responses [1,9,10]. The “Antigen Capsid-Incorporation” strategy is one effort that allows vectors to potentially escape the anti-hAd5 immunity and induce robust specific humoral immune responses by presenting heterologous antigens on hAd5 capsid [11-16].

The well-defined V3 machinery in HIV entry and the fact that V3 is the primary target for neutralizing antibodies [17], have led to major focuses in inhibiting HIV binding/entry via targeting V3 domains. These include developing anti-HIV drugs [18,19], evaluating antiviral activities of multivalent anionic porphyrins [20], bacterial lipopolysaccharide [21], or Epap-1 [22], and the anti-V3 immune response investigations [23-26]. Early study demonstrated that the cyclic V3-loop-related HIV-1 conjugate vaccines elicited neutralizing antibodies [27]. Two V3-based vaccine phase I trials were completed in the 1990s. Of which, the HIV-1 Octameric V3 Peptide Vaccine (Clinical Trials.gov Identifier: NCT00000775) was proven to be safe and able to induce humoral and cell-mediated immune responses [28]. The other vaccine (HIV-1 C4-V3 Polyvalent Peptide) trial (Clinical Trials.gov Identifier: NCT00001060) was also demonstrated safe and immunogenic [29]. Some V3-specific neutralizing monoclonal antibodies (MAbs) have been well characterized. Anti-gp120 (902) MAb [30] for instance, fully neutralizes HIV-1 LAV strain. 447-52D MAb [31] and HGN194 MAb [32] are more examples representing potent and broadly neutralizing MAbs against V3. Notably, the Shimada group reported that Ad5 vector presenting V3 in HVR5 induced cell-mediated V3-specific immune responses in mice with pre-existing immunity against Ad5 [33]. All data highlight the importance of V3 as target for broadly neutralizing antibodies and vaccine development.

Our work is a logical extension of other V3-based vaccine strategies and our ability to insert the ELDKWAS sequence of HIV gp41 in HVR1 of Ad5 hexon using the “Antigen Capsid-Incorporation” strategy [34]. Our question regarding anti-V3 immune response is, could hAd5 vector properly display V3 in the HVR1 region, and trigger efficient V3-specific humoral immune response? Characterizations were undertaken to understand the stability and viability of recombinant hAd5 vectors, the V3 display capability on adenoviral capsid, as well as the V3 antigenicity and immunogenicity.

## Results

### Ad vector construction and characterization

**Figure 1:**
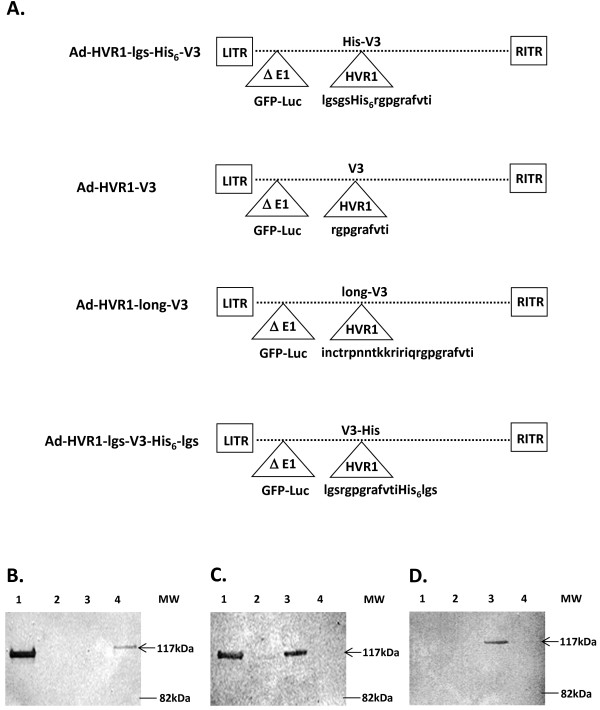
**Construction and validation of four versions of hAd5-based V3 vectors. A)** Construction schematic of four versions of hAd5-based V3 vectors. Four V3 antigens (His-V3, V3, long-V3 and V3-His) were genetically inserted into the HVR1 locale of Ad hexon, using the strategy of “Antigen Capsid-Incorporation”. The E1 region of all four vectors was replaced with GFP-Luc. **B)** After the vector construction and rescue, four vectors were validated for His presentation via western-blot analysis, with the use of anti-penta-His MAb. Line 1, Ad-HVR1-lgs-His_6_-V3; line 2, Ad-HVR1-V3; line 3, Ad-HVR1-long-V3 and line 4, Ad-HVR1-lgs-V3-His_6_-lgs. **C)** V3 presentation was also validated via western-blot analysis, with the use of anti-gp120 (902) MAb. The vector order was the same as the arrangement in Figure 1B. **D)** Anti-IIIB-V3-21 MAb was also used to validate the V3 presentation in the vectors. The vector order was the same as the arrangement in Figure 1B.

Four versions of recombinant Ad5 vectors were constructed and rescued. They were Ad-HVR1-lgs-His_6_-V3, Ad-HVR1-V3, Ad-HVR1-long-V3 and Ad-HVR1-lgs-V3-His_6_-lgs (Figure 1A). All vectors contain the same short V3 sequence (rgpgrafvti, HIV-1 strain IIIB, located in aa 318–327, also named I10 peptide [35]), two of which also contain spacers prior to or after the I10 peptide (Figure 1A). Physical titers and infectious titers were determined, leading to the calculation of VP/IP ratios. A normal VP/IP ratio of unmodified Ad ranges from ~10-30 [34]. We observed normal to slightly increased VP/IP ratios for Ad-HVR1-lgs-His_6_-V3 (ratio of 37) and Ad-HVR1-long-V3 (ratio of 63), when compared to the Ad vector (ratio of 31) (Table 1). These ratios suggested that the insertion of lgs-His_6_-V3 or long-V3 had minimal effects on the stability and infectivity of Ad vectors. Contrarily, the insertion of a short peptide V3 or lgs-V3-His_6_-lgs in the HVR1 strongly affects the stability and infectivity of vectors Ad-HVR1-V3 (ratio of 3.5 × 10^5^) or Ad-HVR1-lgs-V3-His_6_-lgs (ratio of 1.9 × 10^5^) (Table 1).

**Table 1:** Virological properties of vectors

**Modified vectors**	**VP**	**Infectious Particles (IP)**	**VP/IP**
Ad	3.1 × 10^12^ VP/ml	1.0 × 10^11^ IP/ml	31
Ad-HVR1-lgs-His_6_-V3	3.0 × 10^12^ VP/ml	7.9× 10^10^ IP/ml	37
Ad-HVR1-V3	1.4 × 10^12^ VP/ml	4.0 × 10^6^ IP/ml	3.5 × 10^5^
Ad-HVR1-long-V3	1.9 × 10^12^ VP/ml	3.0 × 10^10^ IP/ml	63
Ad-HVR1-lgs-V3-His_6_-lgs	5.5 × 10^11^ VP/ml	2.8 × 10^6^ IP/ml	1.9 × 10^5^

All vectors are serotype and hexon 5-based.

Western-blot was used to validate whether all V3 insertions could be displayed in the Ad5 HVR1. Whereby, anti-penta-His MAb, anti-gp120 (902) MAb (targeting a portion of the V3 epitope, rgpgrafvti) and anti-V3 IIIB MAb (targeting a portion of the V3 epitope, inctrpnntkkririq) were used respectively to detect His_6_ and V3. Results showed that anti-penta-His MAb detected higher levels of His_6_ in Ad-HVR1-lgs-His_6_-V3 than in Ad-HVR1-lgs-V3-His_6_-lgs (Figure 1B). V3 was detected by anti-gp120 (902) MAb both in Ad-HVR1-lgs-His_6_-V3 and Ad-HVR1-long-V3, rather than in Ad-HVR1-V3 or Ad-HVR1-lgs-V3-His_6_-lgs (Figure 1C). Anti-V3 IIIB MAb detected V3 display only in the vector Ad-HVR1-long-V3 (Figure 1D), since only Ad-HVR1-long-V3 contains the specific V3 epitope to anti-V3 IIIB MAb (Figure 1A). The above data suggested that Ad-HVR1-lgs-His_6_-V3 retained both His_6_-specific and V3-specific antigenicity, and Ad-HVR1-long-V3 possessed V3-specific antigenicity.

### V3 could be properly exposed on Ad surface

**Figure 2:**
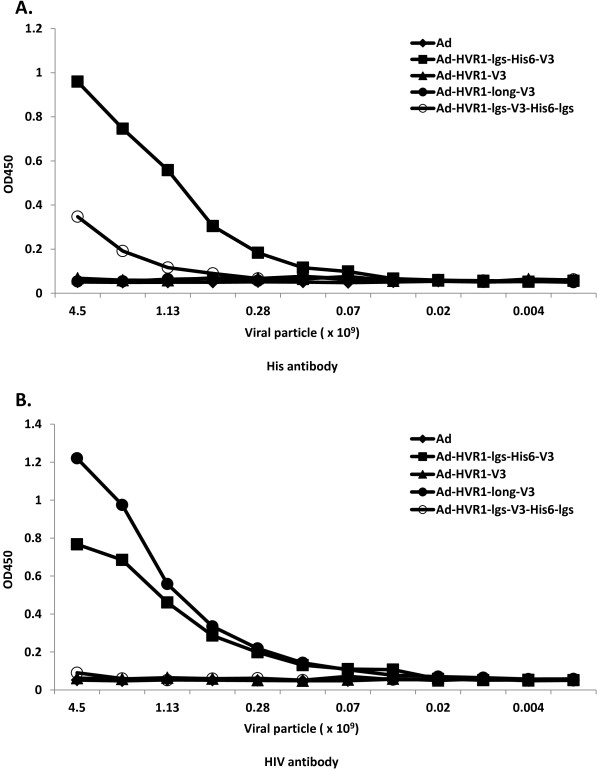
**Evaluation of both the His and V3 exposure capabilities on the capsid surfaces of four versions of hAd5-based V3 vectors.** Whole virus ELISA was employed for this purpose. In this method, four versions of rescued vectors were coated in the wells of ELISA plates at various concentrations, starting from 4.5 × 10^9^ VP/well, and serially diluted at 1:2, until an ending concentration of 4.0 × 10^6^ VP/well. **A)** His exposure was evaluated separately in the vectors: Ad (diamond), Ad-HVR1-lgs-His_6_-V3 (square), Ad-HVR1-V3 (triangle), Ad-HVR1-long-V3 (dark circle) and Ad-HVR1-lgs-V3-His_6_-lgs (blank circle). **B)** V3 exposure was evaluated separately in the same five vectors, with the same order arrangement in Figure 2A. The values were expressed as the means from two replicates.

ELISA can detect antigens without denaturing their advanced structure. By virtue of this advantage, we sought to investigate whether the four versions of V3 antigens and His_6_ could be exposed on the Ad hexon surface, in a way similar to the native formation of corresponding antigens. Results indicated that His_6_ was detected by the anti-penta-His MAb, with higher levels in the vector Ad-HVR1-lgs-His_6_-V3, and much lower levels in the vector Ad-HVR1-lgs-V3-His_6_-lgs (Figure 2A). Anti-gp120 (902) MAb recognized V3 in both vectors Ad-HVR1-lgs-His_6_-V3 and Ad-HVR1-long-V3, whereby the latter showed higher binding capacity (Figure 2B). We speculated that vectors Ad-HVR1-lgs-His_6_-V3 and Ad-HVR1-long-V3 could properly expose V3 on Ad hexon surface in a configuration similar to the native V3 formation. Ad-HVR1-lgs-His_6_-V3 could also properly display His_6_ on the Ad surface.

### V3 in Ad5 HVR1 could be neutralized by a V3-specific antibody

**Figure 3:**
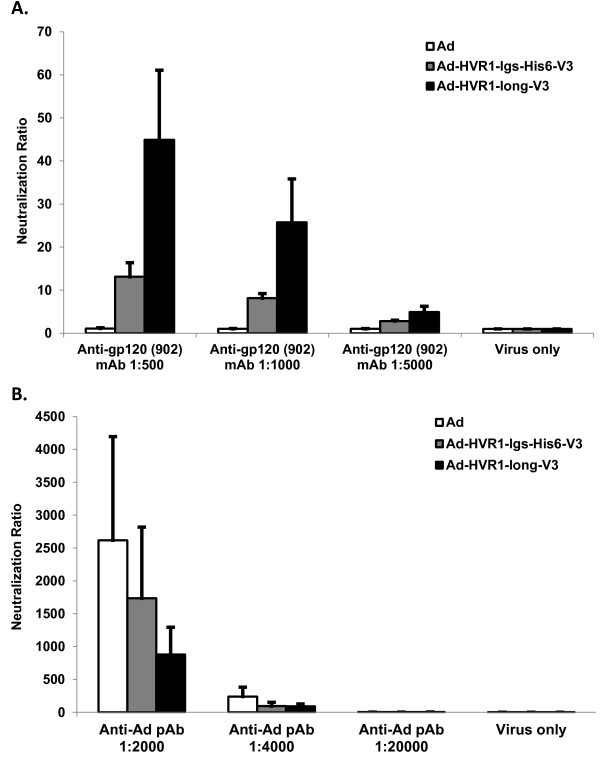
**Neutralization of three Ad vectors by anti-gp120 (902) MAb or anti-Ad PAb. A)** To determine whether the V3-presenting vectors could be neutralized by the anti-gp120 (902) MAb, MAb was diluted into three working concentrations (1:500, 1:1,000 and 1:5,000) and incubated with Ad (negative control), Ad-HVR1-lgs-His_6_-V3 and Ad-HVR1-long-V3, respectively. Then the mixtures were added onto HeLa cells, followed by incubation for 2 hours. The infectious titer was 5 IP/cell. Samples were harvested at 24 hours post-infection (h.p.i.) and subjected to luciferase signal quantification by Luciferase Assay System Kit (Promega). The data were presented as neutralization ratios. **B)** To determine whether the V3-presenting vectors and Ad vector could be neutralized by the anti-Ad PAb, PAb was diluted into three working concentrations (1:2,000, 1:4,000 and 1:20,000), and incubated with Ad, Ad-HVR1-lgs-His_6_-V3 and Ad-HVR1-long-V3, respectively. Samples were harvested at 24 h.p.i. and subjected for measurement of luciferase signal, and the data were presented as neutralization ratios. The values were expressed as the mean ± standard deviation, representing five independent replicates.

To confirm that our vectors present V3 in the most native configuration and thus produce similar V3-specific antibodies in an *in vivo* system, we performed *in vitro* neutralization analyses with gp120 (902) MAb. Statistical analyses comparing any single V3-presenting Ad vector between the antibody-treatment groups and vector only group illustrated that there were significant differences, when the antibody was diluted at 1:500 and 1:1,000, as *p* < 0.001 and *p* < 0.01, respectively, which suggested that both V3-presenting Ad vectors were neutralized with anti-gp120 (902) MAb (Figure 3A). The neutralization ratios illustrated that Ad-HVR1-long-V3 were much higher neutralized (Figure 3A). Analyses comparing the Ad vector between any antibody-treatment group and the vector only group illustrated no significance, suggesting that Ad vector was not neutralized with the HIV antibody (Figure 3A). These data confirmed that Ad-HVR1-lgs-His_6_-V3 and Ad-HVR1-long-V3 could display V3 similar to the native V3 configuration, at least in the anti-gp120 (902) MAb binding site.

As system control, we performed neutralization analyses with anti-Ad polyclonal antibody (PAb) to determine whether the two V3-presenting vectors could be neutralized. Statistical analyses comparing any single vector between the antibody-treatment groups and vector only group illustrated that there were significant differences, only when antibody was diluted at 1:2,000, as *p* < 0.001, which suggested that all the three vectors were neutralized with the anti-Ad PAb, with Ad neutralized the most (Figure 3B).

### Mice immunization triggered V3-specific binding antibodies

**Figure 4:**
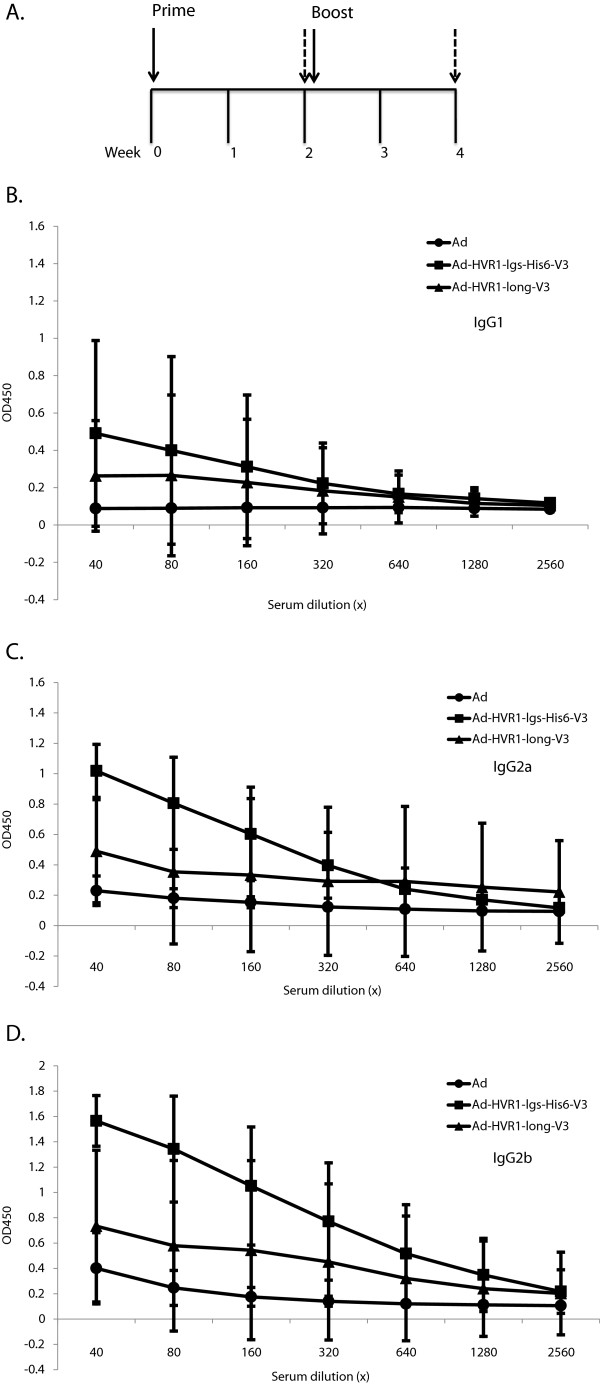
**Induction of V3-specific humoral immune response after immunization. A)** Schematic of “prime-boost” regimen in C57BL/6 mice. Mice were injected with Ad, Ad-HVR1-lgs-His_6_-V3 and Ad-HVR1-long-V3, respectively. The solid lines with arrow denote the time of prime and boost, the dotted lines with arrow denote the time of bleeding post injection. **B)** Sera-based isotype ELISA was employed to determine the levels of V3-specific IgG1 isotype induced in the sera post-boost with vectors Ad, Ad-HVR1-lgs-His_6_-V3 and Ad-HVR1-long-V3. **C)** Sera-based isotype ELISA was employed to determine the levels of V3-specific IgG2a isotype induced in the sera post-boost with vectors Ad, Ad-HVR1-lgs-His_6_-V3 and Ad-HVR1-long-V3. **D)** Sera-based isotype ELISA was employed to determine the levels of V3-specific IgG2b isotype induced in the sera post-boost with vectors Ad, Ad-HVR1-lgs-His_6_-V3 and Ad-HVR1-long-V3. The values were expressed as the mean ± standard deviation, representing eight independent replicates.

The “prime-boost” regimen was employed with Ad vector as control to highlight the potential specific immune responses triggered by V3. The 2-week intervals between prime and boost or between injection and bleeding (Figure 4A) were sufficient enough to trigger antigen specific binding antibodies [34,36].

All sera from post-boost bleeding were first analyzed with V3 peptide, using an isotype ELISA kit to detect three isotypes (IgG1, IgG2a and IgG2b). As predicted, the sera from the Ad group showed baseline signals for all tested isotypes (Figure 4B, C and D). Compared to the Ad group, the Ad-HVR1-lgs-His_6_-V3 group had no significance regarding the presence of IgG1, since *p* > 0.05 (Figure 4B); but the Ad-HVR1-lgs-His_6_-V3 group did show significant signals with respect to both IgG2a (Figure 4C) and IgG2b (Figure 4D). Statistical analyses demonstrated in the IgG2a ELISA that the *p* values (comparison between Ad-HVR1-lgs-His_6_-V3 and Ad groups) corresponding to the sera dilutions ranging from 40× to 320× were <0.0001, 0.0027, 0.0478 and 0.2011, respectively. Statistical analyses also demonstrated in the IgG2b ELISA that the *p* values corresponding to the sera dilutions ranging from 40× to 320× were <0.0001, 0.0003, 0.0049 and 0.0262, respectively. The above two sets of statistical analyses together with the OD450 nm values suggested that Ad-HVR1-lgs-His_6_-V3 triggered higher levels of V3-specific IgG2b response than the V3-specific IgG2a response. Another group (Ad-HVR1-long-V3) showed slightly higher signals in IgG1 (Figure 4B), IgG2a (Figure 4C) and IgG2b (Figure 4D), when compared to the Ad group. Statistical analyses showed no significance between groups of Ad-HVR1-long-V3 and Ad. The long-V3 peptide was also employed in the sera-based ELISA, which confirmed that Ad-HVR1-long-V3 triggered slightly higher binding antibodies without significance compared to the Ad group (data not shown).

## Discussion

The “Antigen Capsid-Incorporation” strategy is advantageous compared to transgene expression because it allows immune boosting of a given antigen after further administration [1,3,34]. Our unpublished findings are that hAd5 could accommodate insertions of up to 57 amino acids in HVR2, up to 77 amino acids in HVR5, and up to 80 amino acids within HVR1. We demonstrated the hAd5 with multivalent incorporation of ELDKWAS within HVR1 and His_6_ within HVR2 or HVR5 [34]. This study utilized hAd5 with the “Antigen Capsid-Incorporation” strategy to generate V3-based HIV-1 vaccine, due to the structure and dominant immunogenicity of V3. V3 contains conserved domains like I10 peptide targeted by cross-clade neutralizing antibodies (NAbs) [37]. This provides a blueprint for designing V3-based vaccines that could elicit cross-clade NAbs. We generated hAd5-based vectors displaying the I10 peptide in HVR1 of hexon, with Ad-HVR1-lgs-His_6_-V3 and Ad-HVR1-long-V3 showing normal VP/IP ratios (Table 1). The Shimada group generated a hAd5-based vector (Ad-V3GFP) carrying the I10 peptide in HVR5 of hexon, but failed in detecting V3 display [33]. The V3 detection in our Ad-HVR1-V3 vector also failed (Figures 1C and 2B), which might be related to improper I10 peptide folding. We speculated that a longer V3 peptide or V3 peptide linked with spacers might help the V3 display. Western-blot detected V3 in Ad-HVR1-lgs-His_6_-V3 and Ad-HVR1-long-V3, but not in Ad-HVR1-lgs-V3-His_6_-lgs (Figure 1C, D). Similarly, whole virus ELISA detected high V3 signal both in Ad-HVR1-lgs-His_6_-V3 and Ad-HVR1-long-V3, but not in Ad-HVR1-lgs-V3-His_6_-lgs (Figure 2B). The above data suggested that longer peptide may help in V3 exposure and antigenicity; a proper spacer-linked peptide may also help V3 exposure and antigenicity, but it might depend on the spacer linkage sites. The importance of introducing proper spacers to the V3 peptide will be further investigated via cryo-electron microscopy analysis. Hence, we were the first to not only generate hAd5-based V3 vectors by insertion in HVR1, but also detect V3 exposure on hAd5 capsid.

Only specific antibody triggered by a given antigen, which has mostly similar structural arrangement to the native configuration of the same antigen, can efficiently target and function on certain infectious pathogens. In this aspect, we need to evaluate the V3 configuration on hAd5 capsid. Neutralization assay illustrated that both V3-presenting vectors were neutralized by the anti-gp120 (902) MAb (Figure 3A). This supported data from the whole virus ELISA that the anti-gp120 (902) MAb bound to Ad-HVR1-lgs-His_6_-V3 and Ad-HVR1-long-V3 (Figure 2B). These data suggested that both vectors Ad-HVR1-lgs-His_6_-V3 and Ad-HVR1-long-V3 could display V3 configuration similar to the native V3 structure. Ad5-specific dominant NAbs appear to be directed largely against the hexon HVRs [38-40]. HVR1 is the largest loop among all the HVRs. However, exchanging just HVR1-HVR3 is insufficient to completely circumvent Ad5 pre-immunity [40]. Based on these facts, our neutralization results with the anti-Ad PAb could mean that since Ad contains native hexon, it was maximally neutralized. Whereas, the V3-presenting vectors lack a portion of HVR1, and the anti-Ad PAb targets multiple sites in hexon. Therefore, the V3-presenting vectors were neutralized to a lesser degree (Figure 3B), which could possibly contribute to Ad5 pre-immunity being moderately circumvented.

The I10 peptide is well known as an H-2D_d_ restricted CD8^+^ CTL specific epitope [41,42], but it also activates specific humoral immune responses [43]. Our immunization assays illustrated that V3-specific binding antibodies were significantly triggered by Ad-HVR1-lgs-His_6_-V3, rather than Ad-HVR1-long-V3, with IgG2a and IgG2b as the dominant isotypes (Figure 4). This indicated that spacer-linked V3 peptide might help present V3 outward to facilitate the immunological recognition, leading to the enhanced V3 immunogenicity when V3 is within HVR1 of Ad5 hexon. And this indication is consistent with previous findings that spacer-linked peptides incorporated into adenovirus hexon protein further improved peptide specific immunogenicity [33,44,45]. Th1 cells activate the down-regulation of IgG1 and up-regulation of IgG2a [46]; Th2 cells up-regulate IgG1 or IgE, but down-regulate other subtypes [47]; while Tregs stimulate productions of IgG2b or IgA [48]. Our results regarding IgG isotype switching suggested that Th1 and Tregs possibly contribute to the V3-specific IgG2a and IgG2b productions, in the mouse model. The investigation of V3-specific binding antibodies is the focus of this paper. We would investigate the V3-specific CD8+ CTL response in the future with class I MHC/I10 peptide tetramer.

Since the mice immunization triggered high magnitudes of V3-specific binding antibodies, we would transition Ad-HVR1-lgs-His_6_-V3 to other animal models for more stringent evaluations. Guinea pig and rabbit models have been employed to investigate NAbs against HIV [49-51]. The well-conserved “gpgr” motif in the V3 of Ad-HVR1-lgs-His_6_-V3 is critical for HIV neutralization [52], and is highly recognized by 447-52D MAb [53]. The comparatively conserved “pgrafvti” motif in the V3 of Ad-HVR1-lgs-His_6_-V3 is highly recognized by anti-gp120 (902) MAb. 447-52D MAb neutralizes a subset of clade B viruses (~45%) [53]. Anti-gp120 (902) MAb neutralizes the majority of HIV-1 clade B strains [30,54]. Therefore, we proposed that Ad-HVR1-lgs-His_6_-V3 might elicit NAbs against V3 in guinea pig or rabbit models; and immunization with Ad-HVR1-lgs-His_6_-V3 might protect against some HIV-1 clade B strains. However, a study showed that the epitope targeted by anti-gp120 (902) MAb may be partially masked, which would impair the neutralizing abilities [55]. The V3 masking in its native configuration on HIV virion is no small task to conquer. Therefore, more studies are needed to overcome this potential hurdle.

## Conclusions

Overall, this study demonstrated that Ad-HVR1-lgs-His_6_-V3 using the “Antigen Capsid-Incorporation” strategy could not only correctly display the V3 of HIV-1 gp120, but also effectively trigger V3-specific humoral immune responses. This “proof-of-concept” Ad-HVR1-lgs-His_6_-V3 with the above concepts will be our next focuses for developing optimal Ad-based V3 vaccine candidates.

## Methods

### Construction of recombinant Ad5 vectors

To construct four versions of Ad5-based recombinant viral vectors displaying V3 of HIV-1 gp120, four DNA fragments (lgs-His_6_-V3, V3, long-V3 and lgs-V3-His_6_-lgs) were synthesized and sub-cloned into HVR1 (a locale replaced from amino acids 139 to 144) of shuttle plasmid H5/pH5S [45]. The resulting plasmids HVR1-lgs-His_6_-V3/pH5S, HVR1-V3/pH5S, HVR1-long-V3/pH5S and HVR1-lgs-V3-His_6_-lgs/pH5S were digested with EcoRI and PmeI. These resulting fragments containing the homologous recombination regions and the hexon genes were recombined through homologous recombination with a SwaI-digested Ad5 backbone lacking the hexon gene, pAd5/ΔH5 [56]. The recombination was performed in *Escherichia coli* BJ5183 (Stratagene, CA), leading to the identification of positive vector clones, respectively. The E1 regions of all four V3-presenting vectors and Ad vector control were replaced with GFP and luciferase as previously described [45].

### Rescue, purification and titration of recombinant Ad5 vectors

To rescue vectors, the recombinant adenoviral genomes were digested with PacI, and transfected with PolyJet (SignaGen Laboratories) into the Ad5-E1-expressing HEK293 cells. Multi-step large-scale propagations of recombinant Ad5 vectors were performed after the vectors were rescued. To purify the rescued vectors, two-step cesium chloride ultracentrifugation was employed, followed by dialysis against 1x PBS containing 10% glycerol. To titrate the purified vectors, physical titers, expressed as viral particles (VPs) per ml were measured using absorbance at 260 nm. The infectious particles (IPs) per ml were determined by TCID_50_ assay [34].

### Western blot

To analyze both the His_6_ and V3 presentations on viral capsid, recombinant vectors were boiled and resolved on SDS-PAGE gels, followed by transfer and blocking on PVDF membranes. Blotting was performed with anti-penta-His MAb (1:2,000; Qiagen, CA), anti-gp120 (902) MAb (1:1,000; NIH AIDS Research & Reference Reagent Program: 522) [30] and anti-IIIB-V3-21 MAb (1:500; NIH AIDS Research & Reference Reagent Program: 1725), respectively, followed by secondary incubation with HRP-conjugated goat anti-mouse antibody (1:5,000; Millipore, MA). The proteins were detected by using 3’3’-diaminobenzidine tablets (Sigma-Aldrich, MO) [34].

### Mice immunizations

Mice immunizations with vectors (Ad, Ad-HVR1-lgs-His_6_-V3 and Ad-HVR1-long-V3) were performed to determine the V3-specific immunogenicity. Female C57BL/6 mice (6–8 weeks) were intramuscularly immunized with corresponding vector (1 × 10^10^ VP/mouse) at each time-point, with a two-week interval between prime and boost. The University of Alabama at Birmingham Institutional Animal Use and Care Committee approved the use of mice as described herein under the approved protocol number 101109272.

### Whole virus ELISAs and sera-based isotype ELISAs

The whole virus ELISAs were performed as described elsewhere [34], in order to investigate the exposure of V3 and His_6_ on surface of capsid. Briefly, different amounts of vectors were immobilized and blocked. The immobilized vectors were incubated with anti-penta-His MAb (1:2,000) and anti-gp120 (902) MAb (1:2,000) separately, followed by incubation with the HRP-conjugated goat anti-mouse antibody (1:5,000). ELISAs were developed with the SIGMAFAST OPD peroxidase substrate (Sigma-Aldrich, MO) and measured at OD 450 nm.

Sera-based IgG isotype ELISAs were performed to determine the magnitude of V3-specific humoral responses and relative isotype development. Briefly, 10 μM V3 peptide (RGPGRAFVTINLEEEDDD) or long-V3 peptide (INCTRPNNTKKRIRIQ) was coated as above. Sera post-boost were sequentially diluted and incubated for 2 hours. Then goat anti-mouse isotypes, IgG1, IgG2a and IgG2b (1:1,000; Sigma-Aldrich, MO) were incubated, followed by incubation with HRP-conjugated donkey anti-goat antibody (1:5,000). Then ELISAs were measured as above.

### Neutralization assay

To determine whether V3-presenting Ad5 vectors could be neutralized by both the anti-gp120 (902) MAb and anti-adenovirus PAb, HeLa cells were seeded and incubated in 6-well plates. Anti-gp120 (902) MAb (stock at 6.4 μg/ml) was diluted into final working dilutions (1:500, 1:1,000 and 1:5,000). Anti-adenovirus PAb was diluted into final working dilutions (1:2,000, 1:4,000 and 1:20,000). Vectors (Ad, Ad-HVR1-lgs-His_6_-V3 and Ad-HVR1-long-V3) were diluted at 5 IP/cell. Diluted antibodies and vectors were co-incubated for 30 minutes at 37°C. Mixtures were then added to relative wells, followed by 2-hour incubation. Thereafter, complete culture medium was added to form a final 1 ml/well volume. The group of vectors without antibody treatment was considered positive control regarding the luciferase expression. Cells at 24 hours post-infection were harvested in Reporter Lysis Buffer (Promega), and luciferase expressions were quantified on the B12 Luminometer (Berthold Detection Systems) by using the Luciferase Reporter 1000 Assay System (Promega). Data of each vector were presented as neutralization ratios, which were the resultants of luciferase signals of one vector in positive control group divided by the signals of the same vector in individual antibody treatment groups.

### Statistical analyses

Descriptive statistics, such as means and standard deviations, were computed for study variables of interest. Comparisons were performed using analysis of variance followed by the Tukey-Kramer multiple comparisons test. Statistical analyses were performed using SAS. Statistical significance was defined as *p* < 0.05.

## Competing interests

The authors do not have any commercial or other association that might pose a conflict of interest.

## Authors’ contributions

Designed the study: LLG, VK, AK, KF, QLM; performed the experiments: LLG, VK, AK; analyzed the data: LLG, VK, AK, RAO; statistical analyses: RAO; wrote the manuscript: LLG, QLM; Proofed the manuscript: LLG, AK, RAO, KF, QLM. All authors read and approved the final manuscript.
